# Ultrasensitive Lateral Flow Immunoassay of Fluoroquinolone Antibiotic Gatifloxacin Using Au@Ag Nanoparticles as a Signal-Enhancing Label

**DOI:** 10.3390/bios14120598

**Published:** 2024-12-06

**Authors:** Olga D. Hendrickson, Nadezhda A. Byzova, Vasily G. Panferov, Elena A. Zvereva, Shen Xing, Anatoly V. Zherdev, Juewen Liu, Hongtao Lei, Boris B. Dzantiev

**Affiliations:** 1 A.N. Bach Institute of Biochemistry, Research Center of Biotechnology of the Russian Academy of Sciences, Leninsky Prospect 33, 119071 Moscow, Russia; odhendrick@gmail.com (O.D.H.); nbyzova@inbi.ras.ru (N.A.B.); panferov-vg@mail.ru (V.G.P.); zverevaea@yandex.ru (E.A.Z.); zherdev@inbi.ras.ru (A.V.Z.); 2 Department of Chemistry, Waterloo Institute for Nanotechnology, Waterloo, ON N2L 3G1, Canada; liujw@uwaterloo.ca; 3 Guangdong Provincial Key Laboratory of Food Quality and Safety, College of Food Science, South China Agricultural University, Guangzhou 510642, China; shenxing325@163.com (S.X.); hongtao@scau.edu.cn (H.L.)

**Keywords:** gatifloxacin, fluoroquinolone, antibiotic, lateral flow immunoassay, Au@Ag nanoparticles, catalytic enhancement, food safety

## Abstract

Gatifloxacin (GAT), an antibiotic belonging to the fluoroquinolone (FQ) class, is a toxicant that may contaminate food products. In this study, a method of ultrasensitive immunochromatographic detection of GAT was developed for the first time. An indirect format of the lateral flow immunoassay (LFIA) was performed. GAT-specific monoclonal antibodies and labeled anti-species antibodies were used in the LFIA. Bimetallic core@shell Au@Ag nanoparticles (Au@Ag NPs) were synthesized as a new label. Peroxidase-mimic properties of Au@Ag NPs allowed for the catalytic enhancement of the signal on test strips, increasing the assay sensitivity. A mechanism of Au@Ag NPs-mediated catalysis was deduced. Signal amplification was achieved through the oxidative etching of Au@Ag NPs by hydrogen peroxide. This resulted in the formation of gold nanoparticles and Ag^+^ ions, which catalyzed the oxidation of the peroxidase substrate. Such “chemical enhancement” allowed for reaching the instrumental limit of detection (LOD, calculated by Three Sigma approach) and cutoff of 0.8 and 20 pg/mL, respectively. The enhanced assay procedure can be completed in 21 min. The enhanced LFIA was tested for GAT detection in raw meat samples, and the recoveries from meat were 78.1–114.8%. This method can be recommended as a promising instrument for the sensitive detection of various toxicants.

## 1. Introduction

The widespread use of antimicrobial drugs in agriculture and veterinary medicine for the treatment and prevention of animal and poultry diseases can lead to their accumulation in livestock products (milk, meat, eggs, and products based on them) [1,2]. Regular consumption of food contaminated with antibiotic residues can cause serious harm to human health—allergic reactions, dysbiosis, candidiasis, gastrointestinal disorders, hepato-, and nephrotoxicity—and lead to the emergence of persistent antibiotic resistance [3,4]. The development of antimicrobial resistance has now become a global problem [5]. Antibiotic resistance critically reduces the effectiveness of drug therapy, which can lead to death. Therefore, strict control of food contamination with antimicrobial agents is a critical task in ensuring the quality and safety of food products.

Fluoroquinolone (FQ) antibiotics are widely used, fully synthetic antibacterial drugs with a broad spectrum of action, good pharmacokinetic properties, and high penetration into tissues and cells of the macroorganism [6,7,8,9]. FQs are characterized by high activity against a large number of bacterial pathogens—most Gram-negative (*Pseudomonas aeruginosa*, *Haemophilus influenzae*, *Escherichia coli*, *Vibrio cholerae*, *Shigella*, *Salmonella*, *Meningococcus*, *Gonococcus*, *Campylobacter*, *Legionella*, etc.) and some Gram-positive bacteria (pneumococci, staphylococci, enterococci) [9]. The biological action of FQs is associated with the inhibition of two vital enzymes of the microbial cell (DNA gyrase and topoisomerase IV), disruption of DNA replication, and consequently, the death of the pathogen [8,10]. Fluoroquinolones always have a piperazine ring and a fluorine atom in their molecular structure.

Gatifloxacin (GAT) is an FQ of the latest, fourth generation. It is active against microorganisms resistant to penicillins, cephalosporins, aminoglycosides, macrolides, and tetracyclines (there is no cross-resistance between the above antibiotics and GAT) and, therefore, is actively used in veterinary medicine to treat pneumonia, bronchitis, pyelonephritis, urinary tract infections, ENT organs, skin, and soft tissues in animals [11]. Like some other FQs of the latest generation, GAT is classified as a “respiratory”, “anti-pneumococcal” antibiotic and is effective against, among other things, *Mycobacterium tuberculosis* [12,13].

The traditional arbitration method for determining various food contaminants, including antibiotics, is high-performance liquid chromatography with mass spectrometric detection (HPLC-MS) [14,15,16,17,18]. This is an accurate and sensitive analytical tool, which, however, can only be implemented by qualified operators in a stationary laboratory with complex and expensive equipment and special reagents and materials. Among other methods for GAT detection, UV-Vis spectrophotometry [19,20], microbiological assays [21], and electrochemical biosensing [22,23] can be mentioned. The two first methods do not provide high sensitivity (LODs lie in the microgram range). In addition, microbiological determination is rather time-consuming. Sensitive electrochemical biosensors (with a recognizing element other than antibodies) require electrodes with complex detecting surfaces (with molecularly imprinted polymers as artificial receptors in [22] and a CuO:Tb^3+^ nanostructure in [23]).

Enzyme-linked immunosorbent assay (ELISA) based on highly specific antigen–antibody interactions is used for antibiotic detection as well [24,25]. This method is simpler than HPLC and may provide highly sensitive determination but also requires some special equipment and certain skills of the operator. In addition, this approach takes several hours to obtain results.

Screening a large number of samples during in-line control of food contamination with antibacterial drugs requires not only accurate and sensitive but also rapid methods that allow for detection in non-laboratory conditions. Such a method is lateral flow immunoassay (LFIA), based on chromatographic separation and immune interactions occurring on membrane carriers assembled into a multimembrane composite (test strip) [26,27]. The main advantages of the LFIA, in addition to sensitivity and specificity, include ease of testing, low cost of test systems, and a short analysis time (10–20 min). Reagents are pre-applied to the test strip, and the analysis consists of incubating the strip with the sample followed by visual and instrumental assessment of product contamination with antibiotics. All this allows for mass “on-site” testing of food samples without special equipment and additional reagents.

Unlike HPLC, the LFIA does not require complicated multi-step sample preparation before testing. Instead, immunochromatography of liquid samples often needs simple sample dilution with a buffer or is performed on an undiluted sample [28,29,30]. However, when analyzing solid food matrices, an extraction procedure associated with significant sample dilution is often demanded [31,32]. Thus, the sensitivity of the LFIA achieved in a buffer is sometimes insufficient for detecting analytes in real samples. Therefore, the development of approaches aimed at decreasing the analyte limit of detection (LOD) is in high demand. It is possible to reduce the LOD in the LFIA via the label—a colored compound that allows for visualization of zones on the test strip after the analytical procedure and evaluation of the assay results. Label-mediated amplification of the analytical signal in immunochromatography can be attained through post-assay growth of label nanoparticles [33,34,35] or by using label catalytic properties, for example, in the case of nanozymes [36,37,38]. It is known that labels based on noble metals (Au, Ag, Pt, Pd) have oxidoreductase-mimic properties and, accordingly, catalyze the oxidation reactions of substrates of different enzymes including peroxidase [39]. The catalytic reaction results in a colored insoluble product that contributes to the brightness of the bands on the test strip. Thus, the colors of the zones on the test strip become more intense, the colorimetric signal increases, and the LOD can be reduced by varying the concentrations of the immunoreagents. Various core@shell nanoparticles have been employed for signal amplification in LFIAs, with platinum-group metals (Pt, Pd, and Ir) receiving the most attention [40]. They have great potential for practical application, but the main limitation to their widespread use is the high cost of the precursor. The discovery of new, more accessible nanomaterials with enzyme-like activity and their application in bioassays as multifunctional tools will contribute to nanozymology development.

Therefore, in this study, a method for the immunochromatographic detection of the antibiotic GAT with bimetallic nanoparticles consisting of a gold core and a silver shell (Au@Ag NPs) with peroxidase-like properties was developed for the first time. Au@Ag NPs served multiple roles: they acted as carriers for antibodies and as optical and catalytic labels. Ag nanomaterials are known to have high molar extinction coefficients, which give them potential as labels for colorimetric LFIAs. Although nanoparticles with a Au–Ag composition have been reported for colorimetric sensors [41,42,43], to our knowledge, Au@Ag NPs have not yet been used in LFIAs as a marker with either colorimetric or catalytic properties. In our previous work, we obtained a label with such a composition but could not select appropriate conditions for the effective use of its catalytic properties and provide signal amplification to increase the assay sensitivity [44]. In the present work, these difficulties have been overcome, and a mechanism for catalytic signal amplification via Au@Ag NPs is proposed. The following tasks were performed: immunoreagents were obtained and characterized, LFIAs of GAT in traditional (with AuNPs) and enhanced (with Au@Ag NPs) formats were developed and compared, and the enhanced format was tested for the determination of GAT in meat samples.

## 2. Materials and Methods

### 2.1. Reagents and Materials

In this study, GAT (racemic mixture of R-GAT and S-GAT enantiomers), silver nitrate, gold (III) chloride hydrate (HAuCl_4_ × H_2_O), sodium citrate, sodium ascorbate, sodium azide, 1-ethyl-3-(3-dimethyl aminopropyl)carbodiimide (EDC), N-hydroxysuccinimide (NHS), bovine serum albumin (BSA), soybean trypsin inhibitor (STI), ovalbumin (OVA), thyroglobulin (TG), sucrose, 2-morpholinoethanesulphonic acid (MES), tris(hydroxymethyl)aminomethane (Tris), 4-(2-hydroxyethyl)-1-piperazineethanesulfonic acid (HEPES), Tween-20, Triton X-100, 30% hydrogen peroxide, dimethyl sulfoxide (DMSO), dimethylformamide (DMF), 3,3′,5,5′-tetramethylbenzidine (TMB), dextran sulfate (Mw = 500.000), and triethylamine from Sigma-Aldrich (St. Louis, MO, USA) were used. S-GAT was obtained from DAICEL (Shanghai, China). Goat anti-mouse immunoglobulins (GAMI), donkey anti-goat immunoglobulins (DAGI), and rabbit anti-mouse immunoglobulins (RAGI) were purchased from Arista Biologicals (Allentown, PA, USA). GAMI and RAGI conjugated with horseradish peroxidase (GAMI–HRP and RAGI–HRP) were sourced from Jackson Immuno Research Labs (West Grove, PA, USA). Monoclonal antibodies (MAb) against S-GAT were produced using the standard hybridoma technique with S-GAT–BSA conjugate as an immunogen [45].

A chromogenic substrate kit for peroxidase activity based on 3,3′-diaminobenzidine (DAB) was purchased from Servicebio (Wuhan, China). A ready-to-use TMB-based substrate solution was obtained from Immunotech (Moscow, Russia). All other chemicals were of analytical grade (Khimmed, Moscow, Russia). All solutions were prepared with ultrapure water with a resistivity of at least 18.2 MW (Millipore Corporation, Burlington, MA, USA).

### 2.2. Synthesis of GAT–Protein Conjugates as Coating Antigens

As coating antigens, GAT–STI, GAT–OVA, and GAT–TG conjugates were obtained following [46] with some modifications. GAT (2.6 mg) in a glass tube was dissolved in DMF (0.3 mL), and then EDC (2.9 mg) and NHS (1.7 mg) were added. The test tube was tightly capped to avoid solvent evaporation and stirred at room temperature (RT) for 2 h. Then, activated hapten was added dropwise to STI, OVA, and TG solutions (7 mg) in 50 mM sodium carbonate buffer, pH 9.5 (3 mL), containing triethylamine (50 µL). The resulting reaction mixture was stirred for 2.5 h at RT and then overnight at 4 °C. The synthesized conjugates were purified by dialysis against PBS. GAT–protein conjugates’ concentrations were determined spectrophotometrically by assessing the optical density (OD) at 280 nm using a Libra UV-Vis spectrophotometer (Biochrom, Cambridge, UK).

### 2.3. ELISA of GAT

GAT–protein conjugates (1 µg/mL, 100 µL in PBS) were immobilized in microplate wells overnight at 4 °C. Then, the microplate was washed four times with PBS containing 0.05% Triton X-100 (PBST). Next, solutions of GAT (16.67 ng/mL–2.5 pg/mL, 50 µL in PBST) and anti-GAT MAb (0.5 µg/mL, 50 µL in PBST) were added to the wells and incubated for 1 h at 37 °C. After washing the microplate as described above, RAGI–HRP (1:3000 dilution, 100 µL in PBST) was added to the wells and incubated for 1 h at 37 °C. After washing, the activity of the enzyme label was determined. To do this, TMB-based substrate solution (100 µL) was added to the microplate wells and incubated for 10–15 min at RT. The reaction was terminated by adding 1 M sulfuric acid (50 µL), and the OD was measured at 450 nm on a Zenyth 3100 microplate spectrophotometer (Anthos Labtec Instruments, Wals, Austria).

### 2.4. Synthesis of AuNPs and Au@Ag NPs

Two preparations of AuNPs (of larger and smaller diameters) were synthesized by HAuCl_4_ reduction with sodium citrate [47]. To obtain larger/smaller AuNPs, 1.0/1.0 mL of 1% HAuCl_4_ solution was added to 97.5/96 mL of deionized water and heated to boiling. After that, 1.5/3 mL of 1% sodium citrate solution was added and stirred while boiling for another 25/20 min. The mixtures were cooled. The obtained AuNPs (OD_520_ = 1) were stored at 4 °C for at least 1 month.

Au@Ag NPs were synthesized following the strategy described in [45]. AuNPs of the smaller diameter were separated from the excess of the citric acid by centrifugation at 12,000× *g* for 20 min and redispersed in an equal volume of ultrapure water. Then, AuNPs (8 mL) were mixed with a 70 mM sodium ascorbate solution (250 µL) and intensively stirred for 30 s. Next, 1.2 mM AgNO_3_ solution (750 µL) was added using a peristaltic pump at a rate of 50 µL/min (i.e., for 15 min). After that, the reduction of silver nitrate was performed for another 20 min with agitation. The synthesized Au@Ag NPs were stored in the dark at 4 °C for at least 1 month.

### 2.5. Characterization of Nanoparticles

To characterize the dimensional parameters of labels, transmission electron microscopy (TEM) was implemented on a JEM-100C microscope (JEOL, Tokyo, Japan). UV-Vis spectra were registered using a Libra S50PC spectrophotometer (Biochrom, Cambridge, UK). When interpreting the spectral data, simulation of the absorption spectra by the online instrument available at NanoComposix (https://nanocomposix.com/pages/mie-theory-calculator (assessed on 22 October 2024)) was used. Dynamic light scattering (DLS) measurements were performed on Zetasizer Nano ZS 90 (Malvern, UK).

The elemental analysis of Au@Ag NPs was performed using energy-dispersive X-ray spectroscopy (EDX) and electron energy loss spectroscopy (EELS). For this, Au@Ag NPs were centrifuged at 12,000× *g* for 10 min and redispersed in ultrapure water. An aliquot (20 µL) of the obtained solution was placed on an ultra-thin carbon-coated copper grid (Ted Pella Inc., Redding, CA, USA) and incubated at RT for 30 min. Characterization of the Au@Ag NPs was conducted using a transmission electron microscope JEOL JEM-F200 (Jeol, Tokyo, Japan) equipped with EDX and EELS modules. Mapping of Au and Ag was performed in EELS mode with a step size of 0.5 nm. The resulting data were analyzed using Digital Micrograph 3.5 software from Gatan Inc. (Pleasanton, CA, USA).

### 2.6. Conjugation of AuNPs and Au@Ag NPs with GAMI

Au@Ag NPs and AuNPs were conjugated with antibodies as follows [44,48]. The excess of the ascorbic acid in Au@Ag NP solution was separated by centrifugation at 15,000× *g* for 15 min. After that, Au@Ag NPs were redispersed in an equal volume of ultrapure water. Before conjugation, the pH of AuNP and Au@Ag NP solutions was brought to 9.0 with 0.2 M K_2_CO_3_. GAMI concentrations for the conjugation with AuNPs and Au@Ag NPs were determined using the technique described in [48]. Antibodies were dialyzed against 10 mM Tris-HCl, pH 9.0, and added to AuNPs and Au@Ag NPs (8 μg/mL in both cases). After 2 h incubation at RT and agitation, BSA was added (to obtain 0.25% solution) and stirred in the same conditions for another 15 min. The synthesized GAMI–AuNP/GAMI–Au@Ag NP conjugates were separated by centrifugation (at 12,000/16,000× *g* for 20/20 min, respectively). Sediments were redispersed in 2.0 mM of Tris buffer, pH 7.5, containing 0.25% BSA, 0.25% Tween-20, and 1% sucrose to obtain preparations 10-fold more concentrated (by volume) than the initial ones. Both preparations were stored at 4 °C.

### 2.7. Manufacturing of Test Strips

For the production of test strips, a nitrocellulose working membrane (CNPCSS12-L2-H50 with 15 µm pore size) on the plastic support and an AP045 adsorption pad were used (Advanced Microdevices, Ambala Cantt, India). The adsorption pad was glued to the top adhesive layer of the support. Then, two zones—a test zone (TZ) and a control zone (CZ)—were formed on the working membrane by the application of GAT–STI conjugate and DAGI, respectively, using an Iso-Flow dispenser (Imagene Technology, Hanover, NH, USA) with a loading of 0.1 µL/mm. The concentrations of GAT–STI/DAGI were 1/0.5 and 0.5/0.25 mg/mL for AuNP- and Au@Ag NP-based test systems. The membrane multicomposites with the applied immunoreagents were dried overnight at RT and for 2 h at 37 °C. Then, the plastic support was trimmed down to the bottom of the working membrane and the multicomposite was cut into test strips of 3.0 mm width with a guillotine (KinBio, Shanghai, China). The resulting test strips were stored in sealed bags with silica gel for at least 2 months without prejudice to the analytical characteristics of test systems.

### 2.8. Sample Preparation

Raw meat (chicken, turkey, beef, pork, rabbit, and lamb) was purchased from a grocery store for organic food. Meat was certified as free of antibiotics and pesticides. For sample preparation, a procedure proposed by Hendrickson et al. (2021) was performed with slight modifications [49]. Meat samples were homogenized with a household blender. We added 10 mM MES buffer, pH 7.7, containing 1% Tween-20 (MES_TW1_) (5 mL) to minced meat (250 mg) and incubated this with stirring for 30 min at RT. Then, the extracts were sonicated in the ultrasound bath for 15 min at RT and centrifuged at 7000× *g* for 15 min at 4 °C to precipitate the solid matrix. The supernatants were carefully collected, divided into 500 mL aliquots, and stored at −18 °C. After defrosting, extracts were 3-fold diluted by MES_TW1_ and analyzed by the LFIA. Thawed extracts were not re-frozen.

### 2.9. Kinetic Measurements of TMB Oxidation

Kinetic measurements were conducted in triplicate using a Tecan Spark microplate spectrophotometer (Männedorf, Switzerland). The temperature during measurements was maintained at 30 ± 1 °C. Substrate solutions (20 mM sodium acetate buffer, pH 4.0, containing 5 mM TMB and H_2_O_2_ at concentrations ranging from 0 to 1.5 M) were prepared immediately before use. Nanoparticle water solution (0.01 mg/mL by gold, 2 µL) was mixed with TMB (100 µL). The OD at 650 nm was recorded every 5 s for 3 min.

### 2.10. LFIA of GAT with AuNPs

In the AuNP-based LFIA, GAT standard solutions (10–0.05 ng/mL, 25 μL in PBS containing 0.25% Tween-20 (PBS_TW0.25_)) and anti-GAT MAb (3.1 µg/mL, 25 µL in PBS_TW0.25_) were mixed and incubated for 3 min. Then, test strips were immersed in the mixtures, incubated for another 3 min, and taken out. Next, 5.8 μL aliquots of GAMI–AuNPs (with a dilution corresponding to OD_520_ = 4.5) were applied close to the bottom of the working membrane (2–3 mm from the lower edge) and left for 1 min. Finally, test strips were washed by immersing them in PBS_TW0.25_ (50 μL) for 3 min.

After the LFIA, the test strips were taken out, blotted, and scanned using a CanoScan LiDE 90 scanner (Canon, Tokyo, Japan). The coloration of TZ and CZ was assessed by the TotalLab TL120 v2009 software (Nonlinear Dynamics, Newcastle upon Tyne, Great Britain, UK) to estimate the color intensity of the zones in relative units (RU).

### 2.11. LFIAs of GAT with Au@Ag NPs in the Common Mode

In the Au@Ag NP-based LFIA without catalytic amplification (hereinafter named a common LFIA), GAT standard solutions (3.7 ng/mL–0.19 pg/mL, 20 μL in MES_TW1_) or meat extracts and anti-GAT MAb (2.5 µg/mL, 20 µL in MES_TW1_) were mixed and incubated for 3 min. Then, test strips were immersed in the mixtures, incubated for another 9 min, and taken out. Next, 1.5 μL aliquots of GAMI–Au@Ag NPs were applied close to the bottom of the working membrane (2–3 mm from the lower edge) and left for 1 min. Finally, test strips were washed by immersing them in PBS_TW0.25_ (50 μL) for 7 min.

### 2.12. LFIAs of GAT with Au@Ag NPs in the Enhanced Mode

The Au@Ag NP-based LFIA with catalytic amplification (hereinafter named the enhanced LFIA) was carried out as described for the common LFIA except for an additional catalytic stage, in which TMB substrate (0.1 mM sodium citrate buffer, pH 4.0, containing 0.83 mM TMB, 1.76 M H_2_O_2_, and 2 µM dextran sulfate) was added to the TZ (10 μL) and incubated for 3 min at RT. Processing of test strips was performed as described above.

### 2.13. Specificity Assessment

Specificity assessment was carried out with a AuNP-based LFIA. Enrofloxacin (ENR), garenofloxacin (GAR), orbifloxacin (ORB), lomefloxacin (LOM), sparfloxacin (SPA), marbofloxacin (MAR), enoxacin (ENO), pipemidic acid (PIP), nadifloxacin (NAD), tosufloxacin (TOS), rufloxacin (RUF), nalidixic acid (NAL), levofloxacin (LEV), ofloxacin (OFL), cinoxacin (CIN), moxifloxacin (MOX), danoloxacin (DAN), pefloxacin (PEF), clinafloxacin (CLI), ciprofloxacin (CIP), difloxacin (DIF), pazufloxacin (PAZ), sarafloxacin (SAR), flumequine (FLU), and oxolinic acid (OXO), all from Sigma-Aldrich (Saint Louis, MO, USA), were applied as structural analogs of the target analyte GAT. To calculate cross-reactivity (CR), the following formula was used: CR = *IC*_50GAT_/*IC*_50analog_ × 100%, where *IC*_50_ is the concentration at the inflection point of the calibration curve of the detected GAT or its analog.

### 2.14. Evaluation of the Immunoassay Results and Statistical Analysis

Dependencies of the TZ coloration (for the LFIA) or OD_450_ (for the ELISA) (y) versus GAT concentrations (x) were processed by a four-parameter logistic function using OriginPro 9.0.0 SR2 b87 software (OriginLab, Northampton, MA, USA). The instrumental LOD (both in the LFIA and the ELISA) was calculated using the Three Sigma approach (the concentration giving a signal equal to the mean value of a blank sample minus 3 SD; the choice of «minus» resulted from competitive assay formats with inverse dependences between analyte concentration and registered signal) [50]. The working range of the detectable concentrations (WR) was considered the GAT concentration range at which a 20–80% signal decrease from its maximal value was registered. The cutoff (or visual LOD) of the LFIA was estimated as the GAT concentration causing the full loss of TZ coloration (i.e., at the colorimetric signal less than 600–700 RU). All quantitative experiments were performed in triplicate, and the means ± SEs (standard errors) of GAT concentrations were calculated. To assess LFIA reproducibility and repeatability, intra-assay and inter-assay variation coefficients were assessed (n = 6). For the analysis of meat extracts, 5 repeats were conducted for each meat sample.

## 3. Results and Discussion

### 3.1. Obtaining Test System Components and Studying Their Properties

At the first stage of the work, the key reagents of the test system were obtained and characterized: markers and specific antibodies. The immune properties of anti-GAT monoclonal antibodies were studied by the indirect competitive ELISA (icELISA) traditionally used for low-molecular analytes. In this assay configuration, the free analyzed antigen in the sample and the protein-conjugated antigen immobilized in the solid phase competitively interact with specific antibodies. The formed immune complexes are detected using anti-species antibodies conjugated with an enzyme label. To implement the ELISA (and, then, LFIA), conjugates of GAT with three proteins (different from BSA used for the immunogen synthesis) were prepared using the carbodiimide method. A hapten was attached to STI, OVA, and TG at molar ratios of 20:1, 40:1, and 100:1. Spectrophotometric characterization of the obtained conjugates revealed the presence of peaks at 280 nm (typical for proteins) and 330 nm (characteristic of GAT) (Figure S1).

**Figure 1:**
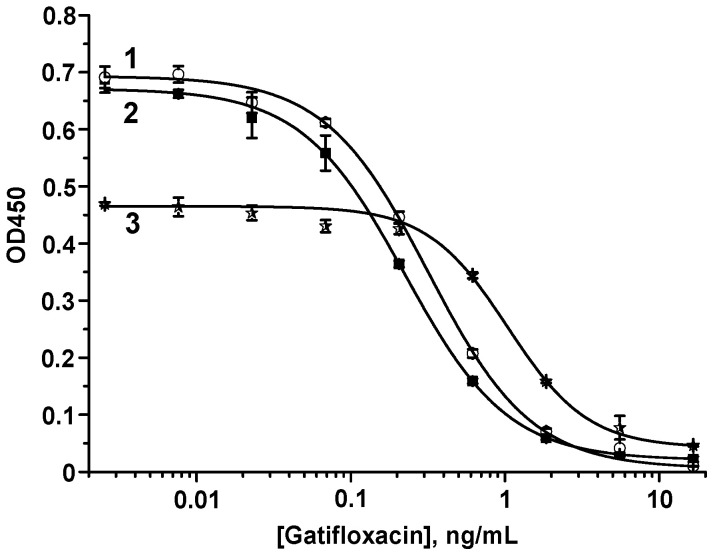
Calibration curves of GAT in the ELISA with GAT–STI (1), GAT–OVA (2), and GAT–TG (3) as coating antigens. The ODs at zero GAT concentrations are 0.71 ± 0.02, 0.69 ± 0.02, and 0.48 ± 0.003 for GAT–STI, GAT–OVA, and GAT–TG as coating antigens.

ELISA was performed using all three coating GAT–protein conjugates: GAT–OVA, GAT–STI, and GAT–TG. According to the obtained competition curves (Figure 1), LODs/WRs were 0.04/0.08–0.7, 0.06/0.1–0.9, and 0.3/0.5–2.4 ng/mL for GAT–OVA, GAT–STI, and GAT–TG, respectively. Thus, the immune properties of the MAb/GAT–protein pairs were confirmed.

**Figure 2:**
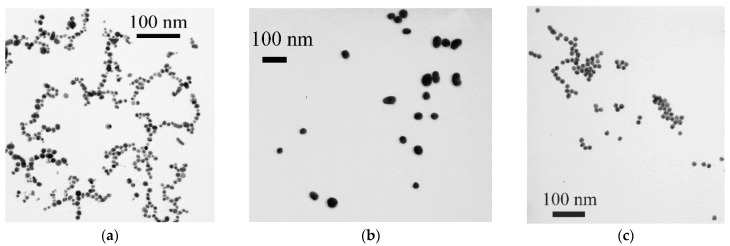
Characterization of labels by TEM: images of AuNPs with an average diameter of 12.1 nm (**a**) and 32.5 nm (**b**) and Au@Ag NPs (**c**).

To develop the LFIA, two types of nanoparticles were prepared: AuNPs and bimetallic nanoparticles consisting of a gold core and a silver shell (Au@Ag NPs). AuNPs were synthesized by reducing gold salt with sodium citrate. Varying the concentration of gold and the reducing agent allows for obtaining gold preparations with different nanoparticle diameters. In this study, two AuNP preparations were synthesized: with a smaller and a larger diameter. According to TEM data, the first preparation contained nanoparticles with an average diameter of 12.1 ± 3.2 nm, with an ellipticity of 1.3 ± 0.3 (64 particles were estimated by dimension) (Figure 2a). Nanoparticles in the second preparation had an average diameter of 32.5 ± 5.3 nm, with an ellipticity of 1.2 ± 0.1 (according to the calculation of 92 particles) (Figure 2b). AuNPs had a spherical shape, and aggregates were not observed in the preparations.

**Figure 3:**
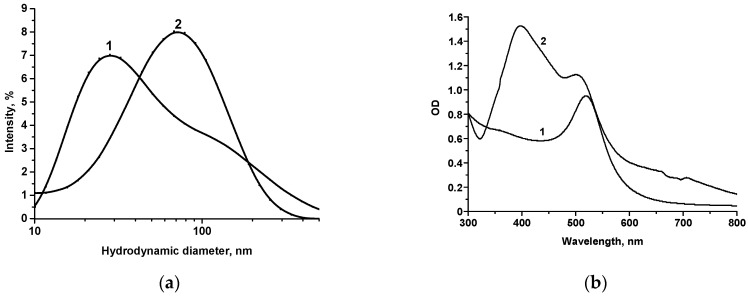
DLS (**a**) and UV-Vis (**b**) spectra of labels: 1—AuNPs with an average diameter of 12.1 nm, 2—Au@Ag NPs.

Spherical AuNPs with an average diameter of 30 nm (in our case, larger ones having 32.5 nm size) are considered the optimal colorimetric label in the LFIA due to their stability and ease of synthesis and conjugation with proteins. They are traditionally used for immunochromatography to form bright red lines on the test strip [51,52]. Based on smaller AuNPs (with a diameter of 12.1 nm), Au@Ag NPs were obtained by reducing silver nitrate with sodium ascorbate on a gold surface according to the technique proposed in our previous study [44]. Electron microscopic characterization of Au@Ag NPs showed the presence of nanoparticles with an average diameter of 14.4 ± 1.2 nm (minimum value—11.7 nm, maximum value—18.5 nm), with an ellipticity of 1.1 ± 0.1 (according to the estimation of 64 particles) (Figure 2c). Another method for label size characterization was dynamic light scattering (DLS) performed on Zetasizer Nano ZS 90 (Malvern, UK). According to the obtained data, the average diameter of small AuNPs (which were utilized to obtain Au@Ag NPs) was ~30 nm (curve 1 in Figure 3a) and of Au@Ag NPs was ~70 nm (curve 2 in Figure 3a). The difference in the dimensional characteristics obtained by the TEM and DLS strategies is explained by the contribution of the hydration shell to the dimensional values. The observed difference is in accordance with data comparing different techniques for the dimensional characterization of nanoparticles reported previously [53,54].

UV-Vis spectra of AuNPs and Au@Ag NPs showed peaks typical of gold (at 520 nm) and silver (400 nm) (Figure 3b). Using Mie theory simulations, we confirmed the presence of two peaks for Au@Ag nanoparticles (Figure S2a). Peak formation at 400 nm was predicted when the thickness of the Ag layer exceeded 0.2 nm. As the Ag shell thickness increased, the intensity of the 400 nm peak also grew. Once the thickness reached 3 nm, the peak at 520 nm disappeared. These simulation results were consistent with the experimental UV-Vis spectra (Figure S2b). Au@Ag nanoparticles synthesized with AgNO_3_ concentrations below 50 µM exhibited only a peak at 520 nm. An increase in AgNO_3_ concentration (50–200 µM) led to the formation of an additional peak at 400 nm. When the AgNO_3_ concentration reached or exceeded 500 µM, only a broad peak at 400 nm was observed. The reported results are in agreement with previously published data [55].

**Figure 4:**
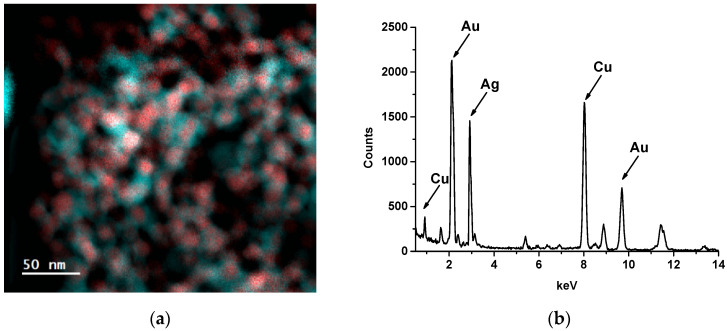
EELS mapping of Au (red objects) and Ag (blue objects) in Au@Ag NPs (**a**), and EDX spectra of Au@Ag NPs (**b**).

Elemental examination of Au@Ag NPs was carried out using EELS and EDX methods. EELS mapping revealed the presence of silver in Au@Ag NPs (Figure 4a). The spatial distributions of Au and Ag confirmed the core@shell structure of the nanoparticles. The EDS spectrum contained peaks characteristic of Au and Ag and, consequently, confirmed the formation of nano-objects composed of these elements (Figure 4b). Copper peaks corresponded to the copper grid used as a solid support for Au@Ag NPs during EDS measurements. High-resolution TEM revealed the core@shell structure of Au@Ag NPs (Figure S3a) with a lattice spacing characteristic of Ag (Figure S3b). The spatial distributions of Au and Ag were nearly identical, which confirmed the thinness (≤2 nm) of the Ag shell (Figure S3c).

In this work, LFIA was carried out in an indirect competitive format, which involves conjugation of the label not with specific but with anti-species antibodies. This format often supplies a gain in assay sensitivity without increasing its duration due to the exclusion of unproductive immune reactions that do not affect the analytical signal [56,57,58]. Conjugates of labels with GAMI were obtained by physical adsorption. The concentration of antibodies for immobilization was selected based on flocculation curves—the dependences of the optical density of labels on the concentration of surface antibodies, which ensures the aggregation stability of nanoparticles. Based on the obtained data (Figure S4), a GAMI concentration of 8 µg/mL was selected to obtain GAMI–AuNP and GAMI–Au@Ag NP conjugates.

### 3.2. LFIA of GAT with AuNPs as a Label

**Figure 5:**
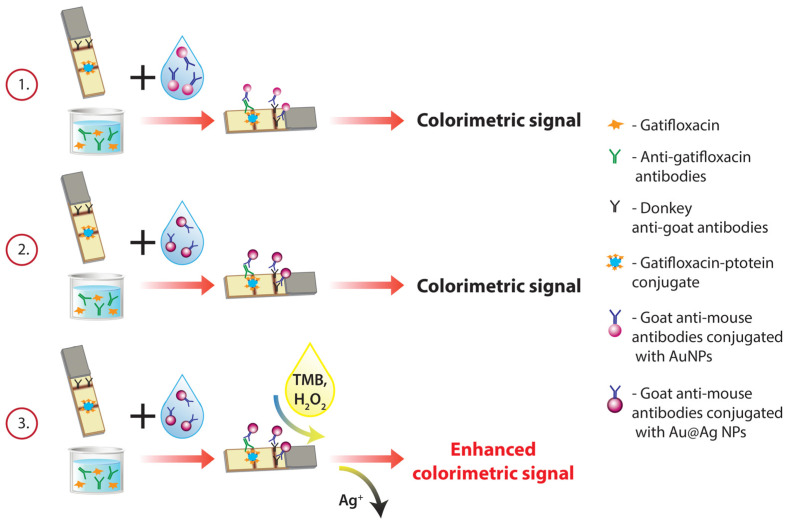
Schemes of the assays performed in this study: AuNP-based LFIA (1), common Au@Ag NP-based LFIA (2), and enhanced Au@Ag NP-based LFIA (3).

To assess the sensitivity gain when using catalytic properties of Au@Ag NPs, the LFIA was performed in three formats: AuNP-based, common Au@Ag NP-based, and enhanced Au@Ag NP-based (schemes 1, 2, and 3 in Figure 5, respectively). The LFIA was carried out in a competitive mode, for which a GAT–protein conjugate and antibodies specific to anti-species antibodies in the labeled conjugate (DAGI) were immobilized in the TZ and CZ on the working membrane, respectively. Free GAT in the sample and GAT–protein conjugate immobilized on the membrane carrier competitively interact with specific antibodies added to the sample. The formed MAb—GAT–protein complexes in the TZ are detected using a colored marker conjugated with GAMI, which interact with MAb. The excess of unreacted labeled GAMI binds to DAGI in the CZ. Thus, with the rise in GAT concentration in the sample, the effect of inhibition of MAb binding to GAT–protein increases and the intensity of the TZ coloration declines until it completely vanishes. Coloration in the CZ should appear in any case, confirming the operability of the test system.

Modern practice of using LFIA test systems includes visual assessment of the presence or absence of visible coloration of the test zone and instrumental estimation of the content of the controlled compound based on recording the coloration intensity for this zone [59,60,61]. Therefore, we consider the instrumental detection mode as a way to make a grounded choice of assay conditions and to demonstrate its capabilities as a quantitative analytical technique.

It should be mentioned that multicomponent labels (such as multimetallic nanoparticles) tend to become stuck on membrane carriers, which was demonstrated in previous works with metal-containing labels [62,63,64]. Therefore, in this study, shortened test strips without a sample and conjugate pads were used (to unify the conditions of AuNP- and Au@Ag NP-based LFIAs). For this, the plastic support was cut to the lower level of the working membrane. Such measures enabled us to significantly reduce the volume of the reaction mixture (from 75–100 µL usually tested with a full-sized test strip to 40 µL) and the duration of the analysis (because the time required for the passage along the shortened strip is significantly reduced). Considering frequent non-specific reactions caused by labels of complex composition, PBS with an increased detergent content (0.25% Tween-20) was used as a reaction medium.

Two schemes of AuNP-based LFIAs were carried out, which differed in the sequence of immune interactions. In the first two-stage scheme, specific MAb and GAMI–AuNP conjugates were added to the GAT-containing sample and incubated. Then, the test strip was dipped in the reaction mixture. In the second variant, the GAT-containing sample was pre-incubated with anti-GAT MAb, and the test strip was dipped in the mixture, incubated again, and removed. An aliquot of GAMI–AuNPs was applied to the lower edge of the working membrane, allowed to soak in, and then the test strip was washed with the buffer. Thus, all interactions were carried out sequentially, and the analysis included four stages.

**Figure 6:**
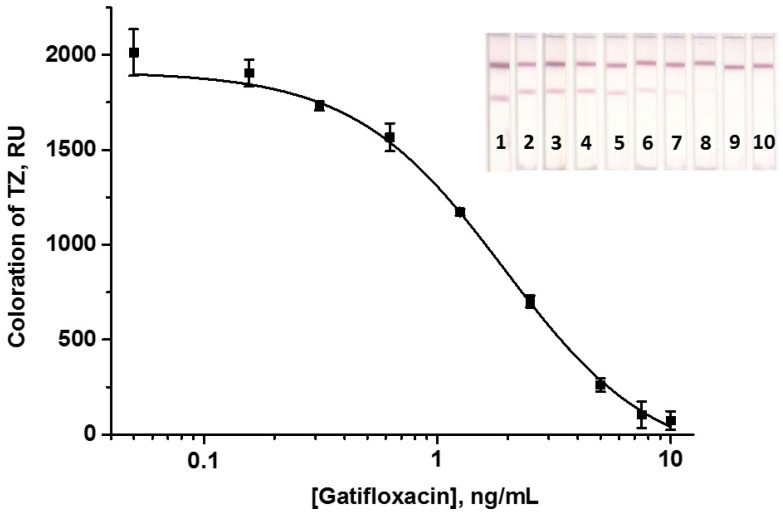
Calibration curve of GAT in the LFIA with AuNPs and the image of scanned test strips. The numbers on the test strips indicate the concentrations of GAT in the samples: 0 (1); 0.05 (2); 0.16 (3); 0.31 (4); 0.62 (5); 1.25 (6); 2.5 (7); 5.0 (8); 7.5 (9); and 10 (10) ng/mL. The signal value at zero GAT concentration is 2030 ± 122 RU.

Both variants were optimized to attain the minimum LOD of GAT. For this, the assay parameters—concentrations of free and adsorbed reagents and the duration of all stages—were varied. The changed parameters and the optimal ones selected for the final analytical procedure are presented in Table S1. GAT–STI was chosen as the protein conjugate immobilized in TZ because it ensured the yielding of the highest signal intensity (Figure S5). Despite more steps in the sequential scheme, its duration was shorter than that of the two-stage LFIA (10 min vs. 13 min). The need for a more complex design was compensated for by the gain in assay sensitivity and colorimetric signal intensity (a comparison of the calibration curves obtained in both modes is presented in Figure S6). Consequently, the sequential ICA variant was selected for further testing (scheme 1 in Figure 5). The GAT calibration curve and images of test strips after AuNP-based LFIA are shown in Figure 6. The following analytical parameters were achieved: instrumental LOD and cutoff of GAT were 0.5 and 7.5 ng/mL, respectively, and WR was 0.7–5.4 ng/mL.

The developed test system was studied for specificity concerning other (fluoro)quinolones. For this purpose, 25 structural analogs of GAT were used: ENR, GAR, ORB, LOM, SPA, MAR, ENO, PIP, NAD, TOS, RUF, NAL, LEV, OFL, CIN, MOX, DAN, PEF, CLI, CIP, DIF, PAZ, SAR, FLU, and OXO. According to the obtained data, GAT-specific MAb cross-reacted with three FQs: LOM (30.1%), CIP (9.8%), and SAR (3%). In other cases, the CR values did not exceed 0.1% (Table S2).

### 3.3. Common LFIA of GAT with Au@Ag NPs as a Label

Au@Ag NP-based LFIA without amplification (scheme 2 in Figure 5) was implemented in the same sequential format as the AuNP-based one. Au@Ag NPs had an orange–brown tint and provided brown bands on the test strip. When switching to another label, re-optimization of the assay system was needed to achieve the best analytical performance. The varied parameters and final assay conditions are presented in Table S1. Foremost, the interaction medium was established. The following buffers were tested: PBS, 10 mM HEPES (pH 7.4), and 10 mM MES (pH 7.7) containing 1% Tween-20 (PBS_TW1_, HEPES_TW1_, and MES_TW1_). It was shown that the maximum level of colorimetric signal is registered when conducting ICA in MES_TW1_ (Figure S7), so this buffer was chosen as the working medium for the assay.

**Figure 7:**
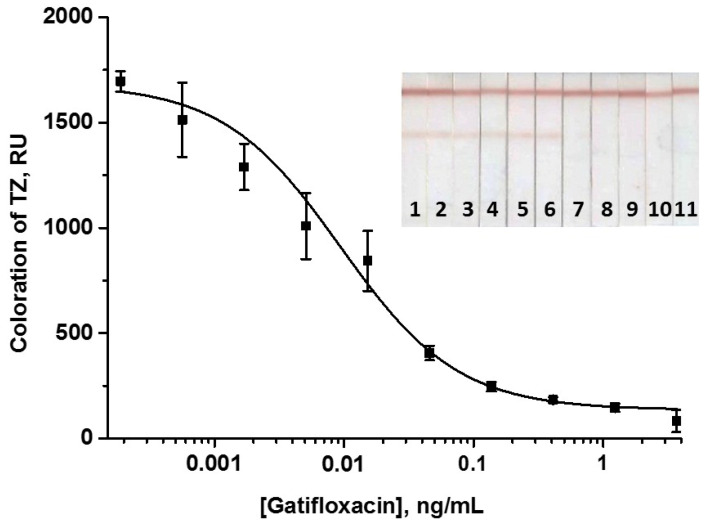
Calibration curve of GAT in the common LFIA with Au@Ag NPs and the image of scanned test strips. The numbers on the test strips indicate the concentrations of GAT in the samples: 0 (1); 1.9 × 10^−4^ (2); 5.6× 10^−4^ (3); 1.7 × 10^−3^ (4); 5.1 × 10^−3^ (5); 0.015 (6); 0.046 (7); 0.14 (8); 0.41 (9); 1.23 (10); and 3.70 (11) ng/mL. The signal value at zero GAT concentration is 1630 ± 54 RU.

When compared with Au@NP-based LFIA, the concentrations of immobilized GAT–STI and DAGI were 2-fold reduced—to 0.5 and 0.25 mg/mL, respectively. The concentration of specific antibodies was also lowered to 2.5 µg/mL, and the volume of the added GAMI–Au@Ag NP conjugate was 1.5 µL. The time of some assay steps was increased compared with Au@NP-based LFIA to guarantee the most effective immune interactions. As can be seen, in AuNP-based and Au@Ag NP-based LFIAs, two stages differed in time: duration of test strip incubation with the sample (3 and 9 min, respectively) and duration of test strip washing (3 and 5 min, respectively). Because complex multicomponent metal labels tend to produce nonspecific sorption on membrane carriers [62,63,64], the time for the indicated steps in the case of the Au@Ag NP-based LFIA was slightly longer (which was not the case for the AuNP-based LFIA, where the gold label moved freely across the membrane). Overall, it increased by only 8 min in the case of the Au@Ag NP-based LFIA, which generally did not affect the assay rapidity. The total LFIA duration was 18 min. The calibration curve of GAT is shown in Figure 7.

The instrumental LOD and cutoff of GAT were 1.4 and 50 pg/mL, respectively, and the WR was 2–40 pg/mL. It should be noted that the obtained values of relative standard deviations are typical for lateral flow immunoassays with nanozyme enhancement [38]. The LFIA with instrumental detection is typically inferior in measurement reproducibility to traditional quantitative methods such as an enzyme immunoassay or chromatography. However, the standard deviation values that characterize the presented development are not critical in limiting its application and practically significant conclusions. Decisions about further actions with the tested food products are made based on a comparison of the detected toxicant concentrations with the officially established maximum residue levels. Following generally accepted practice, after testing by the LFIA, the detected positive samples and some samples from the “gray zone” were additionally controlled with an alternative confirmatory quantitative method [65,66].

Among the directions of further developments that may improve the reproducibility of measurements for instrumental LFIAs with catalytic enhancement, the following approaches are worth noting [35,67,68,69,70]:

- Additional reagents during the formation of binding zones that increase the viscosity of solutions and, consequently, reduce the blur of absorbed reagents;
- Industrial special guillotine cutters for obtaining test strips from sheets with immobilized reagents, allowing for reduced deformation of membrane material at the edges of the strip;
- Reagents for catalytic enhancement being pre-applied to additional membranes and dried before the testing;
- Controlling the liquid flow along the test strips using nanomotors or other reagents, mixing the liquid phase, and preventing interactions in different parts of the binding zone to unify equilibrium conditions.

Manufacturing lateral flow tests—disposable devices with a significant impact of porous heterogeneity and flow variation—into tools for detecting the levels of bound labels causes higher variations from strip to strip as compared with laboratory immunoassays such as ELISA [71]. LFIA optimization allowed for considerable progress in analytical performance compared to the Au@NP-based LFIA. Namely, ~350-fold and 150-fold gains in sensitivity were achieved. We explain this high amplification by the fact that Au@Ag NPs exhibit a higher molar extinction coefficient (ε_380nm_ = 1.5 × 10^9^ M/cm) than AuNPs (ε_520nm_ = 7 × 10^8^ M/cm) [72]. Considering the integrated extinction in the visible region, Au@Ag NPs demonstrate more than a three-fold higher area under the UV-Vis curve (Figure S8). Therefore, Au@Ag NPs are expected to have a lower LOD value.

### 3.4. Mechanism of Signal Enhancement Using Au@Ag NPs

Before the implementation of the enhanced Au@Ag NP-based LFIA of GAT (scheme 3 in Figure 5), it was necessary to confirm the peroxidase-like properties of Au@Ag NPs and their ability to oxidize the peroxidase substrate. For this purpose, kinetic measurements were performed, studying the dependence of optical density during the interaction of Au@Ag NPs with TMB-based peroxidase substrate in the presence of hydrogen peroxide. Au@Ag NPs demonstrated peroxidase properties, oxidizing TMB in the presence of H_2_O_2_ (OD_650_ value increased over time with an increasing concentration of H_2_O_2_, Figure S9). In the absence of H_2_O_2_, the oxidation rate of TMB was negligible, indicating a lack of oxidase activity of Au@Ag NPs. Notably, Au@Ag NPs did not display saturation kinetics typically associated with Michaelis–Menten regularities. As the concentration of H_2_O_2_ increased, a corresponding increase in the reaction rate was observed (Figure S9). As Au@Ag NPs did not exhibit Michaelis–Menten kinetics but oxidized TMB, we hypothesize that the substrate was oxidized by Ag^+^ ions generated through the dissolution of the Ag shell of Au@Ag NPs. In this scenario, the observed signal amplification is attributed to the non-catalytic oxidation of the substrate.

**Figure 8:**
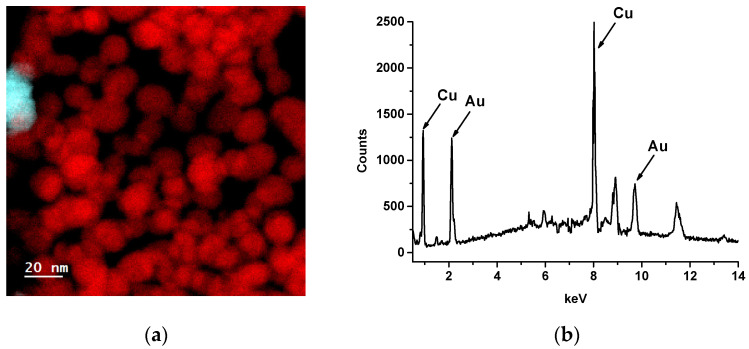
EELS mapping of Au (red objects) and Ag (blue objects) in Au@Ag NPs (**a**) and EDX spectra of Au@Ag NPs after incubation with 10 mM H_2_O_2_ (**b**).

The morphology of the Au@Ag nanoparticles remained similar before and after etching (Figure S10a,b), which was expected given the thin Ag shell (≤2 nm). However, a comparison of the EELS profiles before (Figure S3c) and after the reaction with H_2_O_2_ (Figure S10c) clearly showed the removal of Ag. EELS mapping supported the hypothesis of oxidative dissolution of the Ag layer by H_2_O_2_. The initial Au@Ag NPs displayed high concentrations of silver, and the spatial distributions of Au and Ag confirmed the core@shell structure of the nanoparticles (Figure 8a). However, in the reaction with H_2_O_2_, the silver shell was diluted, resulting in an almost complete absence of Ag in the EELS map (Figure 8a). EDX analysis confirmed the oxidative etching of Ag by H_2_O_2_: the EDX spectrum of the Au@Ag NPs did not display the characteristic Ag Lα line at 2.98 keV (Figure 8b).

**Figure 9:**
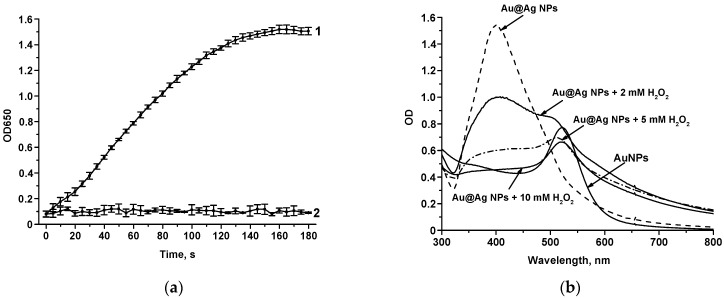
Kinetic curves of TMB oxidation by Ag^+^ ions (1) and AuNPs (2) in the presence of 500 mM H_2_O_2_ (**a**) and spectra of AuNPs and Au@Ag NPs before and after the addition of hydrogen peroxide (**b**).

Furthermore, we confirmed that Ag^+^ ions oxidized TMB while AuNPs exhibited no peroxidase activity (Figure 9a). These results support the proposed signal amplification mechanism, which involves the oxidative etching of Au@Ag NPs, resulting in the formation of bare AuNPs and Ag^+^ ions. UV-Vis spectroscopy confirmed the oxidation of the Ag shell of Au@Ag NPs, facilitating the formation of AuNPs (Figure 9b). Partial oxidation of the Ag shell in the presence of 2 and 5 mM H_2_O_2_ resulted in an absorption peak at 520 nm (characteristic of gold). Oxidation of Ag shell in the presence of 10 mM H_2_O_2_ resulted in almost complete disappearance of the peak at 400 nm (characteristic of silver).

All these findings lead us to propose that Au@Ag NPs cannot be classified as nanozyme catalysts. A defining characteristic of catalysts is that they remain unchanged throughout the catalytic reaction [73]. In contrast, we confirmed the change in the chemical composition of the Au@Ag NPs. Therefore, we may more accurately describe this type of signal amplification as “chemical enhancement” rather than nanozyme enhancement.

### 3.5. Enhanced LFIA of GAT with Au@Ag NPs as a Label

An enhanced LFIA of GAT (scheme 3 in Figure 5) was performed under the conditions selected for the LFIA without amplification. It was critical to choose the composition of the substrate mixture for the analysis. Based on the patterns described above, a higher concentration of Ag^+^ is expected to increase the concentration of oxidized TMB and, consequently, guarantee a higher coloration of the bands on the test strip. Therefore, different concentrations of hydrogen peroxide were added to the substrate mixture containing the unchanged concentration of TMB selected based on our previous studies [74,75]. All substrate mixtures were prepared using 0.1 mM sodium citrate buffer, pH 4.0, containing 0.83 mM TMB. Dextran sulfate was added to the mixture (to a final concentration of 2 µM) because it promoted insoluble product formation, with a violet tone upon TMB oxidation [30]. The concentration of hydrogen peroxide was varied: 17.6 mM (substrate 1), 176 mM (substrate 2), 0.35 M (substrate 3), 0.7 M (substrate 4), and 1.76 M (substrate 5). It was found that with a higher concentration of hydrogen peroxide in the substrate mixture, the signal intensity rises (Figure S11). Further increases in concentration did not lead to an increase in the analytical signal; therefore, substrate mixture 5 was chosen for the enhanced LFIA.

**Figure 10:**
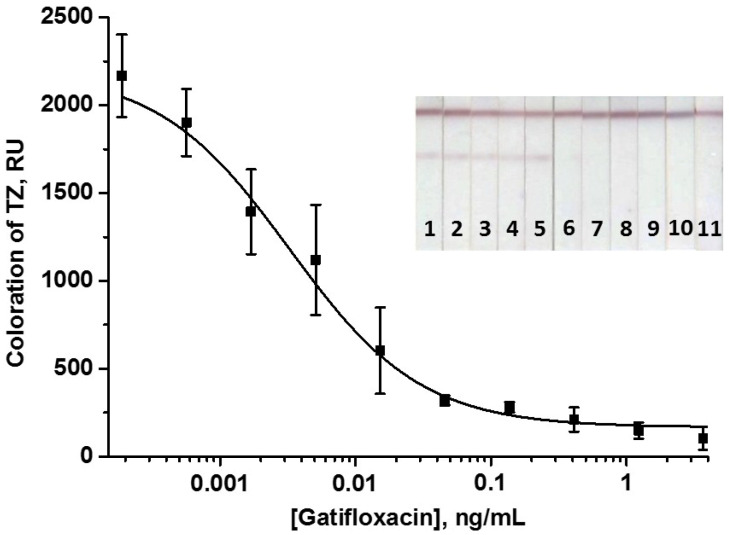
Calibration curve of GAT in the enhanced LFIA with Au@Ag NPs and the image of scanned test strips. The numbers on the test strips indicate the concentrations of GAT in the samples: 0 (1); 1.9 × 10^−4^ (2); 5.6 × 10^−4^ (3); 1.7 × 10^−3^ (4); 5.1 × 10^−3^ (5); 0.015 (6); 0.046 (7); 0.14 (8); 0.41 (9); 1.23 (10); and 3.70 (11) ng/mL. The signal value at zero GAT concentration is 2130 ± 143 RU.

Changing the volume of the substrate mixture applied to the test strips indicated that for consistent distribution over the entire strip surface with coverage of both zones, it was sufficient to apply 10 µL of the substrate to the TZ area. The period of the amplification stage was varied in the range of 1–7 min. It was shown that during the first 3–4 min, the signal grows, and it remains unchanged during further incubation of the test strips with the substrate. Accordingly, the time of the enhanced analysis is 21 min, including an 18 min assay and 3 min catalytic stage. The calibration curve of GAT in the enhanced LFIA is demonstrated in Figure 10.

As can be seen, an enhancement of the colorimetric signal resulted in a shift of WR towards lower analyte concentrations; the instrumental LOD and cutoff of GAT were 0.8 and 20 pg/mL, respectively. Accordingly, 625-fold and 375-fold gains in the assay’s sensitivity were attained compared to the LFIA based on the gold label.

We suggest that the less pronounced concentration-dependent changes in the recorded intensity of coloration in Figure 10 compared to Figure 6 are due to fewer bright and contrasting bands on the test strips provided by Au@Ag NPs compared to AuNPs. When only visual detection can be performed (for example, under out-of-the-laboratory conditions), this is not an obstacle because the presence or absence of visible coloration is used to conclude whether a cutoff concentration has been exceeded of the controlled compound in the tested sample. On the contrary, the concentration dependences of coloration intensities are approximated in cases of instrumental detection with quantitative assessment for differences in the test zones’ coloration.

When comparing the detection sensitivity in the common and enhanced LFIAs with Au@Ag NPs, it is evident that the enhancement process does not supply such a significant gain as when replacing the gold label with a gold–silver one. Based on the regularities described above, nanoparticles with a high loading of Ag are expected to contribute to an increase in the quantity of oxidized TMB. To test this hypothesis, Au@Ag NPs with higher silver loadings were synthesized (i.e., 150, 200, 250, and 500 µM AgNO_3_) and conjugated to GAMI. As shown in Figure S12, the catalytic activity of Au@Ag NPs increases with the concentration of AgNO_3_. However, testing for non-specific interactions showed that incubation of test strips with conjugates based on these labels resulted in colored band formation in the TZ in the absence of GAT and MAb (with the band brightness growing with an increasing silver content, Figure S13). Only the conjugate prepared with 100 µM AgNO_3_ did not exhibit non-specific adsorption on the membrane. Therefore, this conjugate with the lowest silver loading can be used in the enhanced Au@Ag NP-based LFIA. Au@Ag NPs, which are synthesized with a higher concentration of Ag precursor (i.e., a thicker Ag shell) and enable greater signal amplification, could be applied in alternative bioassays, such as ELISA, where non-specific binding to the membrane does not interfere with the assay.

### 3.6. Application of the Enhanced LFIA for GAT Detection in Meat Samples

The developed LFIA with amplification was applied for GAT detection in real matrices. For this, meat samples were selected in which antibiotic residues may have accumulated after the treatment of husbandry animals for various infectious diseases. Raw meat samples of chicken, turkey, rabbit, pork, beef, and lamb purchased from local grocery stores for organic food were used as real samples. Because meat has a complex composition, sample preparation is required to reduce the matrix effect and effectively extract the analyte. Raw meat samples were processed according to the strategy proposed in our previous work [49]. First, spiked raw meat was minced to obtain homogeneous specimens, then GAT was extracted, and the solid part of the matrix was separated by centrifugation.

It was shown that the most effective elimination of the matrix effect requires dilution of the obtained extracts three times by MES_TW1_ before analysis (for comparison, undiluted extracts and extracts diluted 10 times were analyzed). The recoveries of GAT from contaminated meat samples were assessed for GAT concentrations selected from the WR, and the obtained values are presented in Table 1. As can be seen, the enhanced LFIA allows for an antibiotic detection of 78.1–114.8% in meat samples. As a reference method, ELISA was utilized on the same samples. The ELISA application for the comparison was justified by the use of artificially contaminated organic food as test samples, which contained GAT as the only representative of fluoroquinolones and their derivatives. Because the WR of the developed LFIA has a significantly lower concentration in comparison with traditional techniques (see the detailed data in Section 3.7), the recoveries cannot be assessed for the same concentration range in the LFIA and the ELISA. To bring the comparing data closer together, we implemented recovery studies for higher GAT concentrations from the WR of the developed LFIA (i.e., for 12 and 36 ng/g) and lower concentrations from the WR of ELISA (i.e., for 0.96 and 1.44 g/g). The recoveries in the ELISA lay in the range of 73.3–97.3%. Some decrease in the recoveries estimated in the ELISA should be noted. While considering recoveries for LFIA and ELISA, different conditions of competitive interactions should be noted for these two techniques. In the LFIA, residual matrix components are washed out with the lateral liquid flow, and due to this, their inhibiting influence on specific interactions could be reduced. Therefore, for better accuracy of quantitative measurements, additional dilution of real test samples may be needed.

Although recovery studies were implemented with different concentrations of GATI for LFIA and ELISA, the concentrations of antibodies against GATI were unchanged in both cases when varying the concentrations of antigen in the samples. Under the selected conditions of immunochemical interactions, the antibody concentrations were high enough to ensure practically complete antigen binding. This was confirmed by the observed absence of a decrease in recovery for the minimum tested concentrations of GATI. The market location (Moscow, Russia) where meat samples were collected possibly differs from others for regular food intake, but this should not have affected the quality of this study. In further extended validation of the proposed assay, the consideration of naturally contaminated meat samples in combination with HPLC-MS seems reasonable.

When summarizing the data collected in the detection of GATI in real matrices, we can conclude that due to the high sensitivity of the developed LFIA in cases where there is a need for simple control of the MRL excess (in mass screening), significant dilution of samples becomes possible. Such dilution minimizes the potential effects of matrix components on the flow along the test strip, immune interactions, and the recorded coloration. It should be noted that widespread on-site screening control of food toxicants is implemented based on visual detection and leads to the rejection of hazardous food products in accordance with the regulatory stated maximal residue levels and official confirmatory procedures. Instrumentally obtained data on the presence of toxicants in low concentrations do not lead to rejection but provide additional information for monitoring authorities.

### 3.7. Comparison of the Developed LFIA with Other Test Systems

A literature search for the immunochromatographic determination of GAT using GAT-specific antibodies shows that there are no relevant investigations. Works on an immunoassay for GAT based on anti-GAT antibodies are presented in Table 2. As can be seen, all of them (except one study) are microplate analyses of the antibiotic, with various types of detection—colorimetric, chemiluminescent, and even electrochemical [45,76,77,78]. The authors managed to achieve a rather sensitive determination of GAT (LOD of 1 pg/mL was achieved in the case of chemiluminescent detection).

In two other studies, GAT could be detected in ELISA due to the broad specificity of antibodies produced against another FQ—PAZ [79,80]. The authors produced anti-PAZ polyclonal antibodies, which had cross-reactivities of 28.6% [79] and 52.5% [80] towards GAT in a competitive indirect ELISA and allowed for its detection with LODs of 1.43 and 1.72 ng/mL, respectively. Peng et al. (2017) obtained broadly specific MAb using a mixture of NOR and SAR as the hapten. A AuNP-based immunochromatographic analysis was developed, which could detect GAT with an LOD/cutoff of 10/100 ng/mL [81]. A search for the application of bimetallic nanoparticles consisting of a gold core and a silver shell as an immunochromatographic label also revealed the absence of such studies.

Some studies dealt with other analytical methods for GAT detection, as are summarized in Table 2. HPLC remains the main approach for GAT detection in biological matrices and pharmaceutical preparations [14,15,16,17,18]. As mentioned, despite the precision, efficiency, and high resolution, this technique requires laboratories with costly equipment. As can be seen from Table 2, the sensitivity of this method is not always as high (in the nanogram–microgram range) as the results achieved in our study. Spectrophotometry is also applied for GAT detection because of its characteristic absorption peaks in the UV-Vis range [19,20]. The main advantages of this method are simplicity and low cost; however, it also lags in sensitivity (in the microgram range) to immunoanalytical methods including LFIA. High LODs in the electrochemical detection of GAT can be mentioned, but this technique requires special electrochemical biosensors with electrodes with a complex composition [22,23]. The microbiological assay seems an outdated and time-consuming method, which does not meet the requirement of high sensitivity [21].

This work is the first dedicated to the immunochromatographic determination of FQ antibiotic GAT using anti-GAT monoclonal antibodies. A new label—Au@Ag NPs—was used for conjugation with anti-species antibodies and subsequent use in indirect competitive ICA. Because the label had peroxidase-mimic catalytic action, in addition to the common LFIA based on the colorimetric properties of the label, an enhanced assay format was developed. Moreover, a mechanism for such enhancement was proposed and discussed. A supreme sensitivity of GAT detection in the picogram range was achieved. A wide panel of raw meat matrices was used to test the developed test system. The developed test system was rapid, with the whole procedure including sample preparation completed within 1.5 h.

## 4. Conclusions

In this study, the LFIA of gatifloxacin belonging to the fluoroquinolone class of antibiotics was developed for the first time with GAT-specific monoclonal antibodies. A new immunochromatographic label based on bimetallic core@shell Au@Ag NPs having peroxidase-like properties was utilized for common and enhanced modes of the LFIA. A “chemical amplification” mechanism of signal enhancement was proposed. High sensitivity of GAT detection was achieved: LOD and cutoff were 0.8 and 20 pg/mL, respectively. The test system with amplification was successfully tested for GAT determination in several sorts of raw meat. The versatility of the proposed indirect assay scheme as well as the simplicity of label synthesis and modification allow for a recommendation of this approach for the highly sensitive determination of other fluoroquinolones as well as compounds belonging to other classes of food contaminants.

## Figures and Tables

**Table 1:** Recoveries of GAT in the LFIA and ELISA of raw meat samples.

Assay Type			
LFIA			
Meat sample	Added GAT, ng/g	Revealed GAT, ng/g	Recovery ± SD *, %
Chicken	12	12.2 ± 1.2	101.4 ± 10.1
	36	43.6 ± 3.7	121.2 ± 10.2
Turkey	12	13.3 ± 1.5	110.7 ± 12.3
	36	25.8 ± 5.4	71.8 ± 15.1
Rabbit	12	11.8 ± 1.2	98.1 ± 9.9
	36	41.3 ± 3.8	114.8 ± 10.5
Pork	12	13.4 ± 1.6	111.3 ± 13.4
	36	33.9 ± 5.4	94.1 ± 15.1
Lamb	12	12.2 ± 1.2	101.5 ± 9.8
	36	28.5 ± 5.4	79.1 ± 15.0
Beef	12	14.2 ± 1.4	118.4 ± 12.0
	36	36.1 ± 4.1	100.2 ± 11.5
ELISA			
Meat sample	Added GAT, µg/g	Revealed GAT, µg/g	Recovery ± SD *, %
Chicken	0.96	0.86 ± 0.02	89.1 ± 2.6
	1.44	1.33 ± 0.02	90.3 ± 1.3
Turkey	0.96	0.93 ± 0.001	97.3 ± 0.1
	1.44	1.31 ± 0.04	91.3 ± 2.7
Rabbit	0.96	0.86 ± 0.02	89.4 ± 2.1
	1.44	1.24 ± 0.02	86.3 ± 1.3
Pork	0.96	0.82 ± 0.01	85.9 ± 1.5
	1.44	1.20 ± 0.07	83.2 ± 4.8
Lamb	0.96	0.84 ± 0.02	87.6 ± 2.0
	1.44	1.06 ± 0.06	73.3 ± 4.0
Beef	0.96	0.83 ± 0.02	87.1 ± 2.2
	1.44	1.27 ± 0.05	88.2 ± 3.2

* Standard deviation.

**Table 2:** List of studies on GAT analysis.

Immunoassays				
Assay type	Hapten used for antibody production	Sensitivity	Matrix	Reference
icELISA with electrochemical detection	GAT	*IC*_50_ = 8.9 ng/mL	Swine urine	[76]
icELISA with colorimetric detection	GAT	LOD = 0.05 ng/mL	Milk	[77]
icELISA with colorimetric detection	GAT	*IC*_50_ = 4.7 ng/mL	Buffer	[45]
icELISA with chemiluminescent detection	GAT	LOD = 1 pg/mL	Milk	[78]
icELISA with colorimetric detection	PAZ	LOD = 1.43 ng/mL	Buffer	[79]
icELISA with colorimetric detection	PAZ	LOD = 1.72 ng/mL	Milk	[80]
LFIA	NOR and SAR	LOD/cutoff = 10/100 ng/mL	Milk	[81]
LFIA with enhancement	GAT	LOD = 0.8 pg/mL Cutoff = 20 ng/mL	Different kind of raw meat	This study
Other analytical methods				
LC-MS/MS	n/n *	Linear range = 1.56–400 ng/mL	Rabbit tear fluid	[15]
Ultra HPLC	n/n *	LOD = 0.09 ng/mL	Milk, honey	[16]
HPLC	n/n *	LOD = 25 µg/mL	Ophthalmic solution	[17]
Micellar LC	n/n *	Linear range = 5–45 µg/mL	Pharmaceutical formulation	[18]
UV-Vis spectrometric analysis	n/n *	Linear range = 0.075–0.25 µg/mL	Human urine, lake water	[19]
UV-Vis spectrometric analysis	n/n *	Linear range = 4–14 µg/mL	Pharmaceutical formulation	[20]
Microbiological assay	n/n *	Linear range = 4–16 µg/mL	Pharmaceutical formulation	[21]
Electrochemical detection	n/n *	LOD = 2.61 × 10^−15^ M	Real water samples	[22]
Electrochemical detection	n/n *	LOD = 1.2 nM	Milk, human serum, pharmaceutical formulation	[23]

* Not needed.

## Data Availability

The original contributions of this study are included in this article and the Supplementary Materials, and further inquiries can be directed to the corresponding author.

## Supplementary Materials

The following supporting information can be downloaded at https://www.mdpi.com/article/10.3390/bios14120598/s1, Figure S1: UV-Vis spectra of GAT, STI, OVA, TG, and GAT–protein conjugates; Figure S2: Simulated optical spectra for Au@Ag NPs with various Ag shell thicknesses. AuNPs with a diameter of 12 nm were used as a core (a). Experimental optical spectra for Au@Ag NPs with various concentrations of AgNO_3_ used for synthesis (b); Figure S3: High-resolution TEM microphotographs of Au@Ag confirming the core@shell structure of Au@Ag NPs (a) with a lattice spacing characteristic of Ag (b) and EELS profile for Au and Ag (c); Figure S4: Flocculation curves obtained for AuNPs (1) and Au@Ag NPs (2); Figure S5: Signal intensities in the AuNP-based LFIA achieved using different GAT–protein conjugates; Figure S6: Calibration curves of GAT in the sequential (1) and two-stage (2) formats of LFIAs with AuNPs as a label; Figure S7: Images of test strips after incubation with GAMI–Au@Ag NPs in different media (GAT concentration is 0 ng/mL); Figure S8: The relationships between OD_520_ and AuNP concentration (a) and between OD_380_ and Au@Ag NP concentration (b), applied to calculate the molar extinction coefficient and theoretical UV-Vis spectra, showing the areas under the curves, which correspond to the integrated extinction in the visible region (c); Figure S9: Kinetic curves of TMB oxidation by Au@Ag NPs at H_2_O_2_ concentrations of 1500 (1), 1000 (2), 500 (3), 200 (4), 100 (5), 50 (6), and 0 (7) mM; Figure S10: TEM microphotographs of Au@Ag confirming the core@shell structure of Au@Ag NPs after etching (a) with a lattice spacing characteristic of Ag (b) and EELS profile for Au and Ag (c); Figure S11: Images of test strips after incubation with substrate mixtures with different contents of hydrogen peroxide (at GAT concentration of 1.25 ng/mL); Figure S12: Kinetic curves of TMB oxidation by Au@Ag NPs obtained under different concentrations of AgNO_3_ upon synthesis (50–500 µM); Figure S13: Images of test strips after incubation with GAMI–Au@Ag NPs with different levels of silver loading (no GAT or MAb in the sample); Table S1: Parameters varied during optimization of the LFIAs of GAT; Table S2: Specificity of the test system.
