# Supplementary material for: Ultrasensitive Lateral Flow Immunoassay of Fluoroquinolone Antibiotic Gatifloxacin Using Au@Ag Nanoparticles as a Signal-Enhancing Label
Source: Biosensors (Basel). 2024 Dec 6;14(12):598. doi: 10.3390/bios14120598 (PMC11674194; doi:10.3390/bios14120598)
Supplement: Supplementary file 1 [file biosensors-14-00598-s001.zip › biosensors-3302147-supplementary.pdf]

biosensors

Article

Ultrasensitive Lateral Flow Immunoassay of Fluoroquinolone Antibiotic Gatifloxacin Using Au@Ag Nanoparticles as a Signal-Enhancing Label

Olga D. Hendrickson [1], Nadezhda A. Byzova [1], Vasily G. Panferov [1,2], Elena A. Zvereva [1], Shen Xing [3], Anatoly V. Zherdev [1], Juewen Liu [2], Hongtao Lei [3] and Boris B. Dzantiev [1,*]

[1] A.N. Bach Institute of Biochemistry, Research Center of Biotechnology of the Russian Academy of Sciences, Leninsky Prospect 33, 119071 Moscow, Russia; odhendrick@gmail.com (O.D.H.); nbyzova@inbi.ras.ru (N.A.B.); panferov-vg@mail.ru (V.G.P.); zverevaea@yandex.ru (E.A.Z.); zherdev@inbi.ras.ru (A.V.Z.)

[2] Department of Chemistry, Waterloo Institute for Nanotechnology, Waterloo, ON N2L 3G1, Canada; liujw@uwaterloo.ca

[3] Guangdong Provincial Key Laboratory of Food Quality and Safety, College of Food Science, South China Agricultural University, Guangzhou 510642, China; shenxing325@163.com (S.X.); hongtao@scau.edu.cn (H.L.)

* Correspondence: dzantiev@inbi.ras.ru; Tel.: +7-495-954-31-42

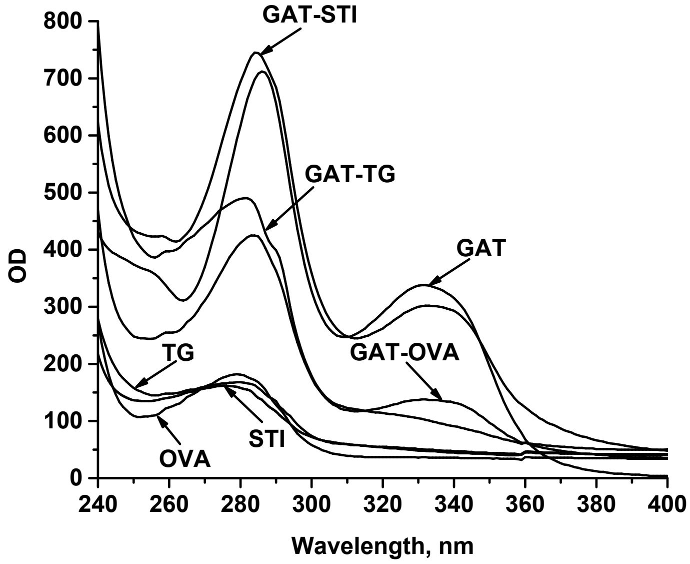

Figure S1. UV-Vis spectra of GAT, STI, OVA, TG, and GAT–protein conjugates.

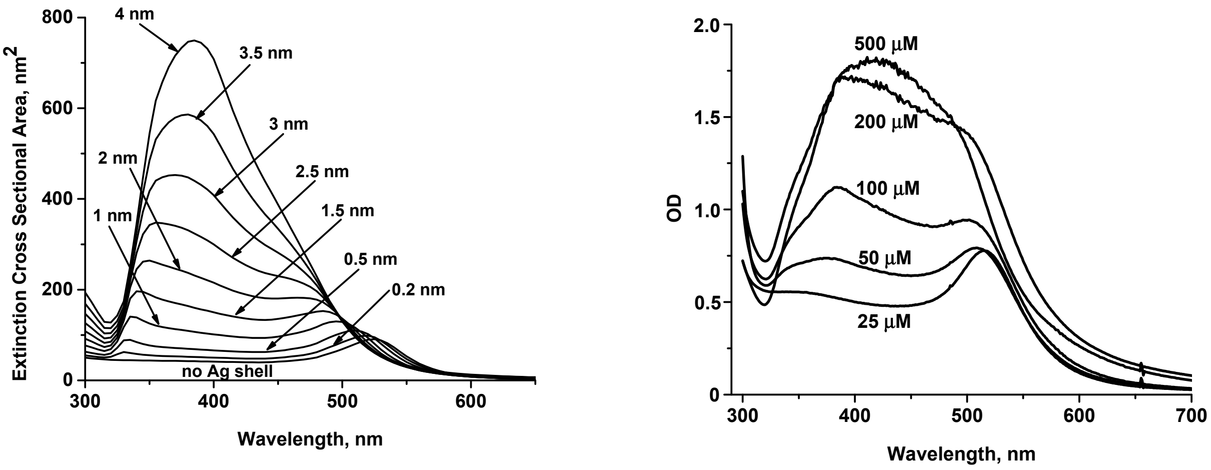

(a) **(b)**

Figure S2. Simulated optical spectra for Au@Ag NPs with various Ag shell thicknesses. AuNPs with a diameter of 12 nm were used as a core (a). Experimental optical spectra for Au@Ag NPs with various concentrations of $AgNO_3$ used for synthesis (b).

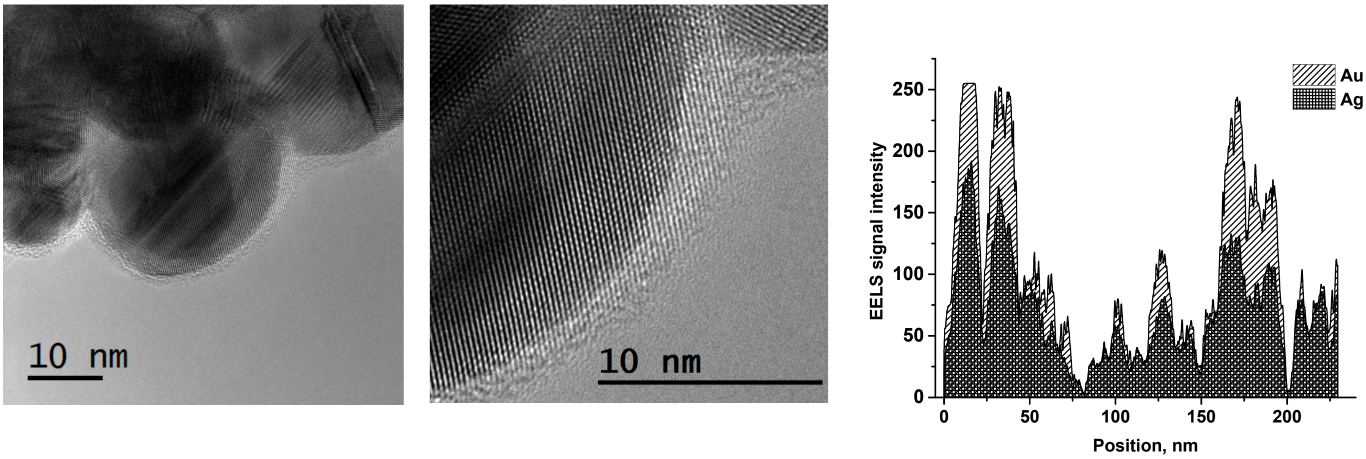

(a) **(b)** **(c)**

Figure S3. High-resolution TEM microphotographs of Au@Ag confirming the core@shell structure of Au@Ag NPs (a) with a lattice spacing characteristic of Ag (b) and EELS profile for Au and Ag (c).

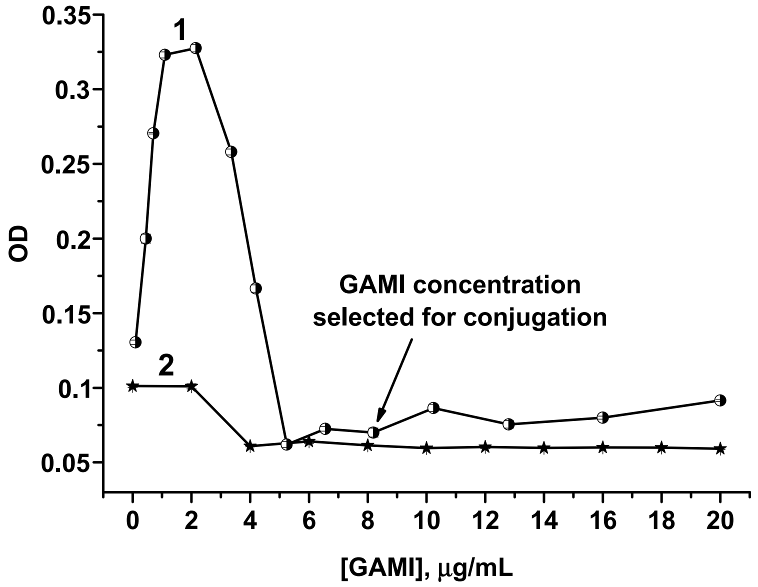

Figure S4. Flocculation curves obtained for AuNPs (1) and Au@Ag NPs (2).

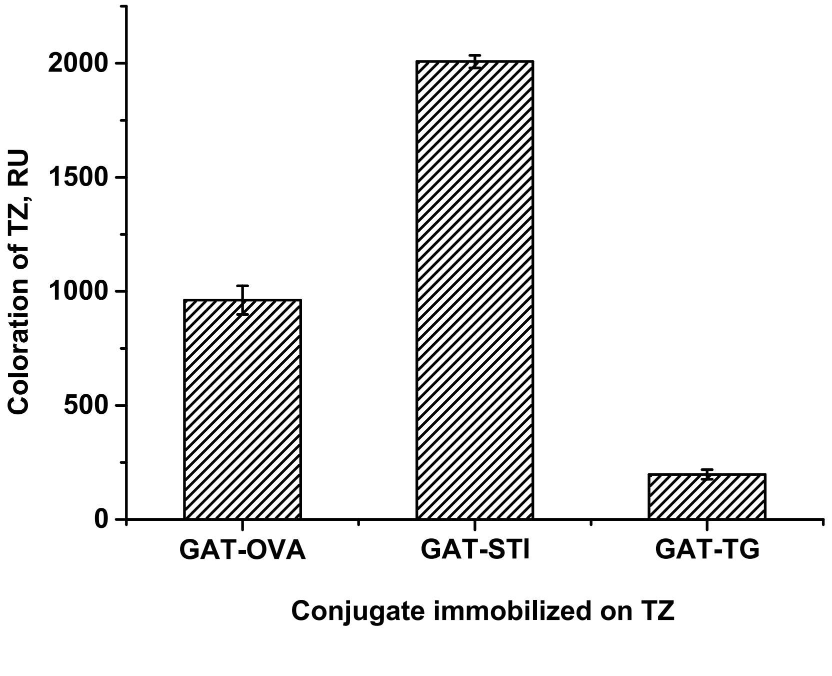

Figure S5. Signal intensities in the AuNP-based LFIA achieved using different GAT–protein conjugates.

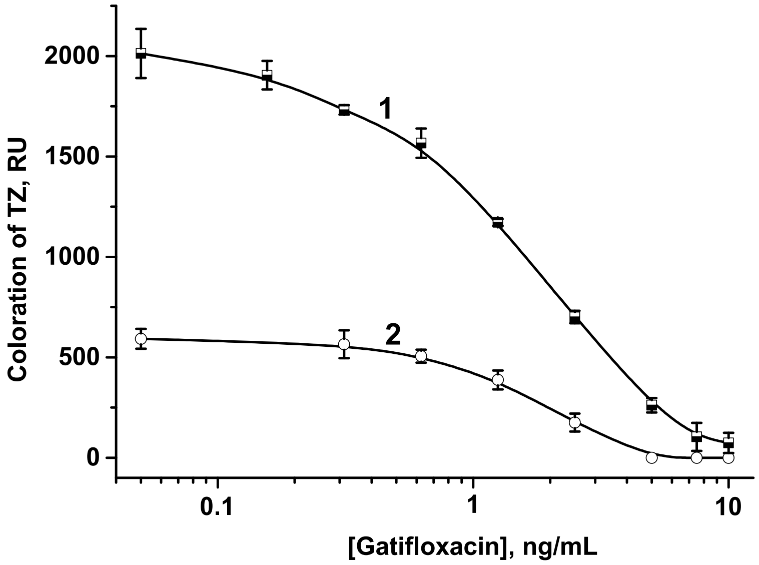

Figure S6. Calibration curves of GAT in the sequential (1) and two-stage (2) formats of LFIAs with AuNPs as a label.

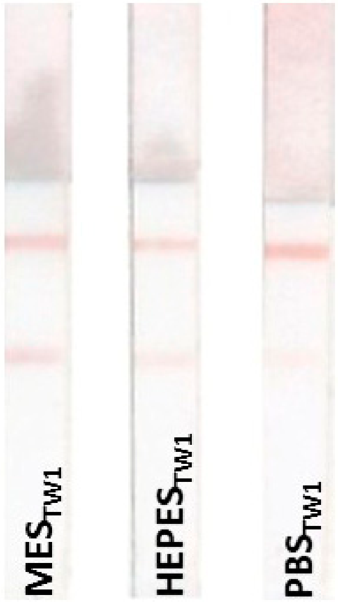

Figure S7. Images of test strips after incubation with GAMI–Au@Ag NPs in different media (GAT concentration is 0 ng/mL).

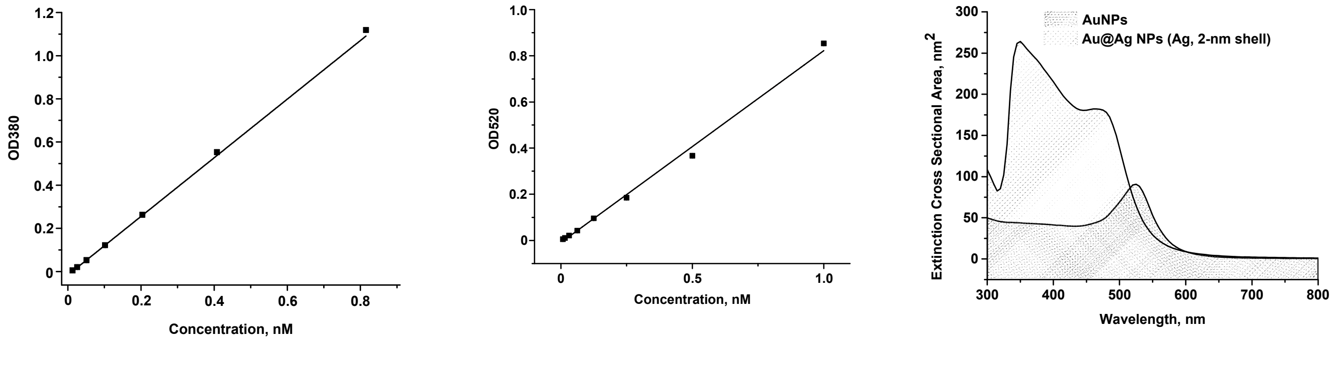

(a) (b) (c)

Figure S8. The relationships between OD_{520} and AuNP concentration (a) and between OD_{380} and Au@Ag NP concentration (b) applied to calculate the molar extinction coefficient and theoretical UV-Vis spectra, showing the areas under the curves, which correspond to the integrated extinction in the visible region (c).

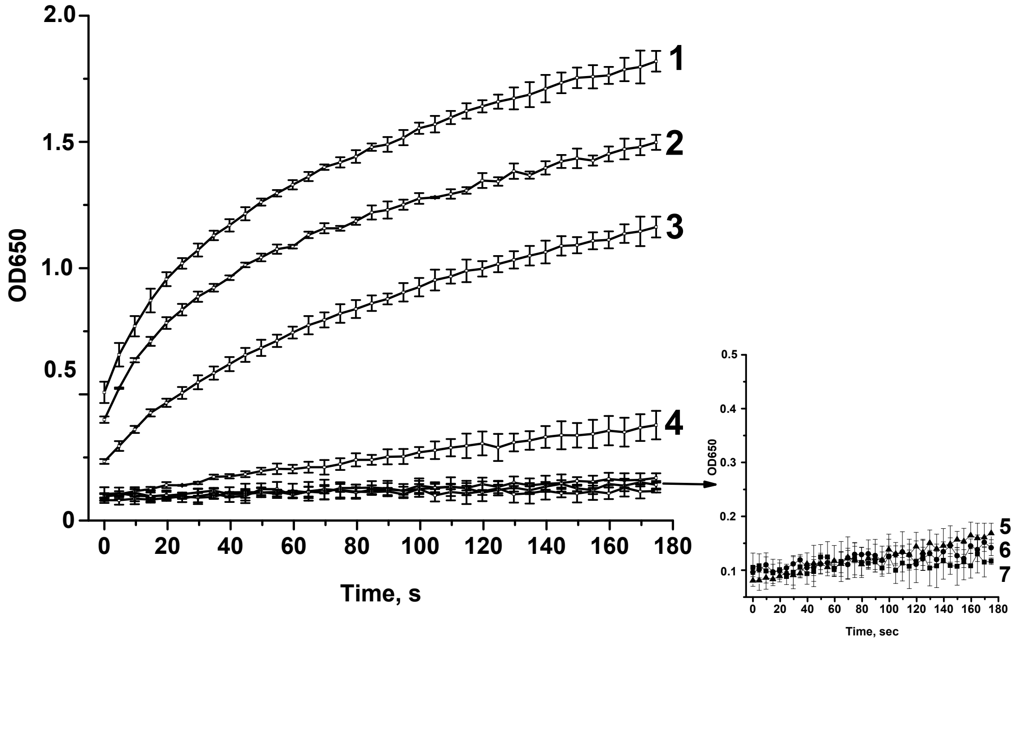

Figure S9. Kinetic curves of TMB oxidation by Au@Ag NPs at H_2O_2 concentrations of 1500 (1), 1000 (2), 500 (3), 200 (4), 100 (5), 50 (6), and 0 (7) mM.

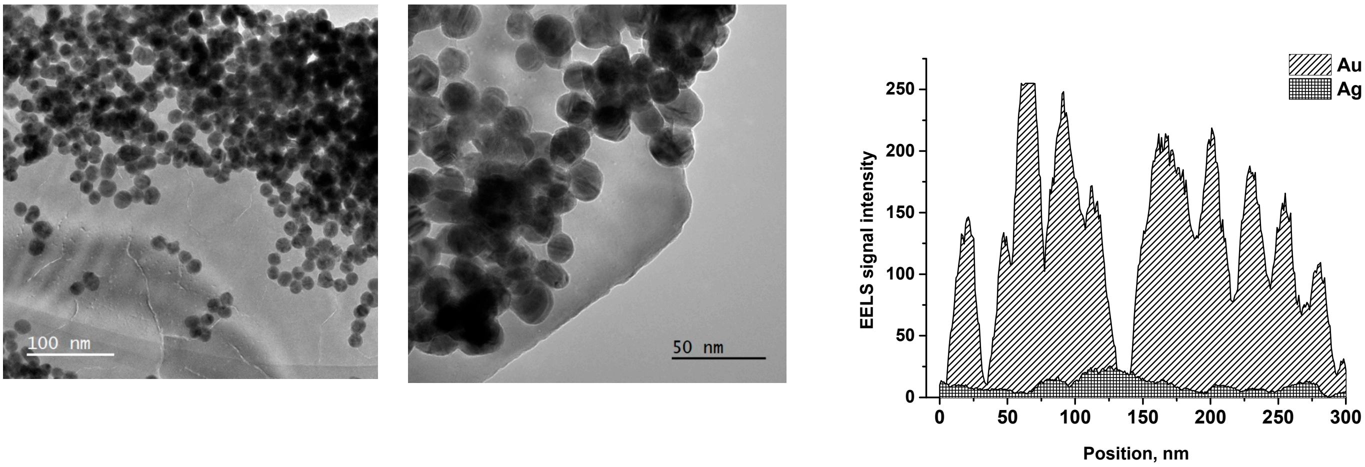

(a) **(b)** **(c)**

Figure S10. TEM microphotographs of Au@Ag confirming the core@shell structure of Au@Ag NPs after etching (a) with a lattice spacing characteristic of Ag (b) and EELS profile for Au and Ag (c).

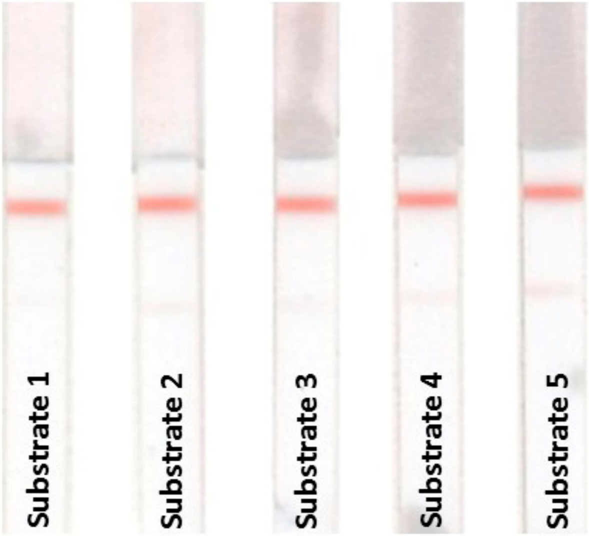

Figure S11. Images of test strips after incubation with substrate mixtures with different contents of hydrogen peroxide (at GAT concentration of 1.25 ng/mL).

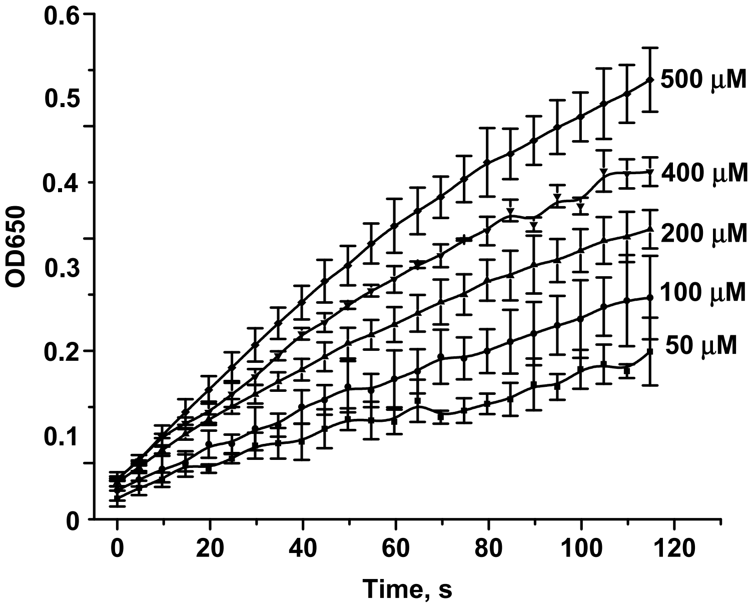

Figure S12. Kinetic curves of TMB oxidation by Au@Ag NPs obtained under different concentrationss of $AgNO_3$ upon synthesis (50–500 μM).

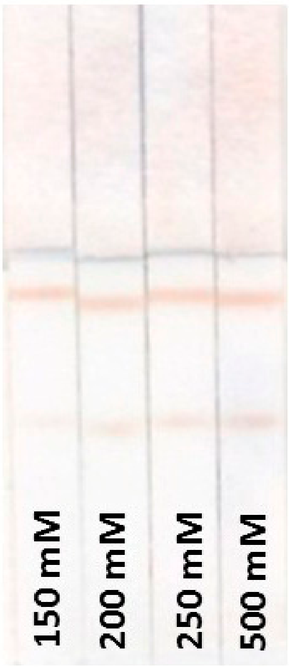

Figure S13. Images of test strips after incubation with GAMI–Au@Ag NPs with different levels of silver loading (no GAT or MAb in the sample).

Table S1. Parameters varied during optimization of the LFIAs of GAT

Parameter	Varied diapason	Selected value
	AuNP-based LFIA	
Immobilized GAT–protein, mg/mL	GAT–OVA; GAT–STI; GAT–TG	GAT–STI
Concentration of the immobilized GAT–STI in the TZ, mg/mL	0.25–2	1
Concentration of the immobilized DAGI in the CZ, mg/mL	0.2–0.6	0.5
Concentration of anti-GAT MAb, μg/mL	0.5–5	3.1
Number of GAMI–AuNPs, μL	1–8	5.8
Duration of pre-incubation, min	1–5	3
Duration of test strip incubation with the sample, min	1–5	3
Duration of test strip incubation with GAMI–AuNPs, min	0.5–5	1
Duration of test strip washing, min	1–5	3
	Au@Ag NP-based LFIA (common and enhanced modes)	
Medium for interactions	PBS_{TW1}; $HEPES_{TW1}$; MES_{TW1}	MES_{TW1}
Concentration of the immobilized GAT–STI in the TZ, mg/mL	0.25–2	0.5
Concentration of the immobilized DAGI in the CZ, mg/mL	0.2–0.5	0.25
Concentration of anti-GAT MAb, μg/mL	0.5–5	2.5
Number of GAMI–Au@Ag NPs, μL	1–8	5.8
Duration of pre-incubation, min	1–5	3
Duration of test strip incubation with the sample, min	1–15	9
Duration of test strip incubation with GAMI–Au@Ag NPs, min	0.5–5	1
Duration of test strip washing, min	1–10	5
Volume of the added substrate, μL (for enhanced format)	1–25	10
Duration of the amplification stage, min (for enhanced format)	1–7	3

Table S2. Specificity of the test system.

**Detected analyte**	***IC₅₀*, ng/mL**	**CR, %**
GAT	1.9	100
ENR	>2000	<0.1
GAR	>2000	<0.1
ORB	>2000	<0.1
LOM	6.1	30.1
SPA	>2000	<0.1
MAR	>2000	<0.1
ENO	>2000	<0.1
PIP	>2000	<0.1
NAD	>2000	<0.1
TOS	>2000	<0.1
RUF	>2000	<0.1
NAL	>2000	<0.1
LEV	>2000	<0.1
OFL	>2000	<0.1
CIN	>2000	<0.1
MOX	>2000	<0.1
DAN	>2000	<0.1
PEF	>2000	<0.1
CLI	>2000	<0.1
CIP	19.4	9.8
DIF	>2000	<0.1
PAZ	>2000	<0.1
SAR	63.3	3
FLU	>2000	<0.1
OXO	>2000	<0.1
